# *Corallodiscus flabellata *B. L. Burtt extract alleviates lipopolysaccharide/D-galactosamine-induced acute liver failure and brain injury by inhibiting oxidative stress, apoptosis, and inflammation

**DOI:** 10.22038/ijbms.2020.45437.10567

**Published:** 2020-11

**Authors:** Benke Li, Mengnan Zeng, Beibei Zhang, Yuxuan Kan, Shenchao Wang, Yangyang Wang, Yuanyuan Wu, Ruiqi Xu, Weisheng Feng, Xiaoke Zheng

**Affiliations:** 1 College of Medicine, Henan University of Chinese Medicine, Zhengzhou 450046, China; 2 The Engineering and Technology Center for Chinese Medicine Development of Henan Province, Zhengzhou 450046, China

**Keywords:** Acute liver failure, Apoptosis, Aquaporin, Brain, * Corallodiscus flabellata *B. L. Burtt, Inflammation, Oxidative stress

## Abstract

**Objective(s):**

*Corallodiscus flabellata *B. L. Burtt (CF) is distributed along liver meridian, with a possible beneficial effect in the progression of acute liver failure. Therefore, the present study investigates the effect of CF extract on rats with acute liver failure.

**Materials and Methods:**

Rats were divided into four experimental groups: Control, Lipopolysaccharide (LPS)/D-Galactosamine (D-GalN) (L/D), Wu Ling Powder + L/D (WLP+L/D) and CF + L/D. Animals were gavage for 7 days, after which all animals except the control group were injected intraperitoneally with LPS and D-GalN to induce acute liver failure. Subsequently, the urine was collected for the next 8 hr, and the liver pathological changes were observed. The levels of alanine aminotransferase (ALT), aspartate aminotransferase (AST), inflammatory factor and oxidative stress-related indicators were measured. The levels of reactive oxygen species (ROS), apoptosis marker in the liver, water content and aquaporin (AQPs) in the brain were detected. The concentration of ions and osmolality of urine and serum were determined.

**Results:**

The results show that CF significantly improved the damage of liver and brain tissue, and reversed the changes of serum ALT, AST, inflammatory factor and Cl-. It modulated oxidative stress-related indicators, reduced the content of ROS, apoptosis markers, water content, the level of Cl- ions and osmolality in the urine and the expression of AQP1, and AQP4 in the brain, and increased the urine output.

**Conclusion:**

It was found that the CF extract could alleviate the L/D induced acute liver failure by regulating the hepatocyte apoptosis and AQPs expression in the brain.

## Introduction

Acute liver failure (ALF) is a serious liver damage caused by a variety of factors, such as chronic alcoholism, exposure to chemical substances, and viral hepatitis. The mortality ratio in ALF is very high, and cerebral edema (CE) and intracranial hypertension (ICH) have long been recognized as the cardinal features and major causes of death in ALF (1). Recent studies have confirmed that the occurrence and prognosis of ALF are closely related to inflammatory response and oxidative stress (2). D-Galactosamine (D-GalN) is a specific hepatotoxic agent specifically metabolized in hepatocytes, which reduces intracellular uracil nucleotide pools, thereby inhibiting RNA and protein synthesis. When administered with low doses of lipopolysaccharide (LPS), animals that are highly sensitive to D-GalN develop fatal liver failure, showing biochemical and metabolic changes similar to ALF. Both ALF and CE currently do not have very effective treatments; hence, targeted development of drugs for these conditions is a very urgent task (3).

*Corallodiscus flabellata *B. L. Burtt, the entire herb of *C. flabellata*, which is also called Yanzhijia and Shiheye etc., is a common herb in the Chinese herbal medicine. It is mainly distributed in the Sichuan, Henan, Yunnan and Tibet provinces. Studies have shown that the chemical constituents in this herb include phenylethanoids, phenylethanoid glycosides and flavonoid glycosides (4). According to the Chinese system of medicine, the extract of *C. flabellata *(CF) is distributed along liver meridian and has the effects of clearing away damp heat, relieving sore venom, promoting blood circulation and relieving pain. People in the Funiu Mountain area of the Henan Province often use it to treat pharyngitis, colds, irregular menstruation, etc. (5). At present, there are no reports on the intervention effect of CF in ALF. In this pursuit, the present study envisages the intervention effect of CF in ALF–induced rats and to explore its effect on CE caused by ALF.

## Materials and Methods

***Experimental drug***

*C. flabellata *B. L. Burtt was collected from the Xixia County, Henan Province, China, and identified by Professor Chen Suiqing, Henan University of Chinese Medicine. A voucher specimen (No.20180467A) was deposited in the Research Department of Natural Medicinal Chemistry, School of Pharmacy, Henan University of Chinese Medicine. Dry herb powder (1.0 kg) was treated three times with boiling 50% ethanol (10 l × 3, 1.5 hr each time) to prepare the extract. Evaporation of the solvent under reduced pressure provided an aqueous extract (155 g). The extracts were dissolved in distilled water before the experiment to obtain a drug solution of 108.5 mg/ml.

Wu Ling Powder Capsule (WLP) (180502) was used as a positive control drug, and was provided by Jiangxi Pinxin Pharmaceutical Co., Ltd, China. LPS (057M4013V) and D-GalN (SLBT9951) were purchased from Sigma (MO, USA).

***Experimental animals***

Male Wistar rats, (6-8 weeks), weighing 200±20 g were obtained from Beijing Vital River Laboratory Animal Technology Co., Ltd., China (Ethical approval reference number: SCXK2016-0011). Animals were housed in cages on a 12-hr light/dark schedule at a controlled temperature (23±1 °C) with free access to food and water at the specific-pathogen-free laboratory of Animal Research Center of Henan University of Chinese medicine. The study was conducted in accordance with the Regulations of Experimental Animal Administration issued by the State Committee of Science and Technology of the People’s Republic of China. The protocol was approved by the Institutional Animal Care and Use Committee of Henan University of Chinese Medical Laboratory Animal Research Center, (Henan, China Approval Number: HACTCM-2019002060-14).

***Experimental method***

Animals were divided into four groups (18 rats in each group) according to the principle of weight balance and randomization: Control group (CON), L/D group, WLP+L/D group (WLP 63 mg/kg, positive control group) and CF+L/D group (CF, 7000 mg/kg). The WLP+L/D and CF+L/D groups were gavage for 7 days in advance, once a day by intragastric administration, while these groups were given the same amount of distilled water per day. After 0.5 hr of the last administration, all animals except the CON group were injected intraperitoneally with LPS and D-GalN (10 μg/kg and 900 mg/kg) to induce an ALF. The CON group was injected with the same amount of normal saline. Then, the animals were placed in metabolic cages to collect urine for the next 8 hr. Subsequently, the animals were anesthetized with 3% isoflurane gas and the blood sampling from the abdominal aorta was performed. Animals were then sacrificed by an overdose of isoflurane, and the brain and liver tissues were collected.

***Histopathological evaluation***

The liver and brain were fixed in freshly prepared 4% paraformaldehyde, embedded in paraffin, and then cut into 5-μm-thick sections. Hematoxylin and eosin (HE) staining was performed as per the standard protocols and images were taken with a microscope (Nikon, Tokyo, Japan). The sections were observed in non-consecutive, free selection 200× or 400× histological fields and the representative images were captured.

***Biochemical indexes assay***

The levels of alanine aminotransferase (ALT, C009-2, Nanjing Jiancheng Bioengineering Institute, Nanjing, China), aspartate aminotransferase (AST, C010-2, Nanjing Jiancheng Bioengineering Institute, Nanjing, China), malondialdehyde (MDA, A003-1, Nanjing Jiancheng Bioengineering Institute, Nanjing, China), glutathione peroxidase (GSH-PX, A005, Nanjing Jiancheng Bioengineering Institute, Nanjing, China), total superoxide dismutase (T-SOD, A001-1, Nanjing Jiancheng Bioengineering Institute, Nanjing, China), sodium (Na^+^, C002, Nanjing Jiancheng Bioengineering Institute, Nanjing, China), potassium (K^+^, C001-2, Nanjing Jiancheng Bioengineering Institute, Nanjing, China) and chlorine (Cl^-^, C003-2, Nanjing Jiancheng Bioengineering Institute, Nanjing, China) in serum were detected. The levels of Na^+^, K^+^, and Cl^-^ in urine were detected as per the instructions in the kit.

***Serum inflammatory factors determination***

BD™ Cytometric Bead Array (CBA) technology was used to detect the levels of inflammatory factors in the rat serum. To 50 μl of serum from each group, 50 μl of interleukin-6 (IL-6, 558305), tumor necrosis factor (TNF, 558309), interleukin-2 (IL-2, 561420), and interleukin-10 (IL-10, 558306) microsphere working solution was added and incubated at room temperature for 1 hr in the dark. Subsequently, 50 μl of the antibody working solution of IL-6, TNF, IL-2, and IL-10 (BD Biosciences, USA) was added and incubated at room temperature for 2 hr in the dark. One milliliter of wash buffer (BD Biosciences, USA) was then added and centrifuged at 300 g for 5 min, the supernatant was discarded, and 300 μl of wash buffer was added and detected at 488 nm wavelength using flow cytometry (BD Biosciences, USA).

***Determination of ROS content in primary liver cells***

The liver tissues of the anesthetized rats from each group were taken, cut and digested with trypsin, and the digestive juice was filtered through a 70 μm filter to obtain a rat primary cell suspension, which was carefully processed according to the instructions of the reactive oxygen species (ROS) detection kit (CA1410, Solarbio Science & Technology Co., Ltd, Beijing, China), and subsequently detected by flow cytometry (BD Biosciences, USA). ROS content was expressed by mean fluorescence intensity (MFI).

***Western blot analysis***

The Western blot analysis was performed to detect the expression levels of AQP1 (ab9566, Abcam, MA, USA), AQP4 (ab15081, Abcam), caspase-3 (ab13847, Abcam), caspase-9 (ab32539, Abcam), Bax (Ab32503, Abcam), Bcl-2 (ab59348, Abcam), and β-actin (ab8266, Abcam). Protein extraction kit (20180822, Beijing Solarbio Science & Technology Co., Ltd, China) was employed to detect and quantify the proteins using a BCA protein assay kit (20180822, Beijing Solarbio Science & Technology Co., Ltd, China). The protein samples were separated by sodium dodecyl sulfate - polyacrylamide gel electrophoresis. Further, 60 μg of each protein sample was loaded into the gel and transferred to a polyvinylidene fluoride (PVDF) membrane. Then, nonfat milk was used to block the membrane for 2 hr at room temperature. The membrane was incubated with a primary antibody AQP1 (1:1000), AQP4 (1:1000), caspase-3(1:1000), caspase-9 (1:1000), Bcl-2 (1:1000), Bax (1:1000), and β-actin (1:2000) overnight at 4 °C and washed with Tris-Buffered saline in Tween-20 (TBST) buffer. Subsequently, this mixture was incubated with a secondary antibody (goat anti-rabbit 925-68071, goat anti-mouse 925-32210, Li-COR, MO, USA) for 1 hr at room temperature. In addition, after the PVDF membrane was washed with antibody using a washing solution, another primary antibody was added to continue the test. The intensity of the proteins was quantified using Odyssey (Clx, Li-COR).

***Brain water content determination***

After the animals were sacrificed, the brain tissue was quickly removed (without brainstem and cerebellum), and the surface liquid was absorbed by filter paper. The electronic balance (Sartorious, Göttingen, Germany) was employed for weighing, and the weight was recorded as wet weight. Further, the tissues were transferred to an oven at 100 °C for 24 hr and re-weighed the dry weight, and the brain water content was calculated as per Equation (1).

Brain water content = (wet weight - dry weight) / wet weight*100%-(1)

***Osmolality determination***

The osmolalities of urine and serum were determined by an Osmometer (OM815; Loser Messtechnik).

***Statistical analysis***

Data was analyzed using SPSS 20.0 (IBM, NY, USA). Statistical significance was assessed in comparison with the respective control for each experiment using one-way analysis of variance, and *P* values less than 0.05 was accepted as significant.

## Results

***Effects of CF on pathological damage of liver tissue and brain tissue***

**Figure 1:**
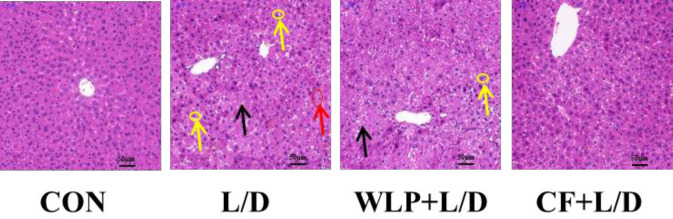
Effect of CF on L/D-induced pathological damage of liver tissue. WLP+L/D group (WLP, 63 mg/kg) and CF+L/D group (CF, 7000 mg/kg) were administered for 7 days, followed by intraperitoneal injection with L/D (10 μg/kg and 900 mg/kg). Yellow arrow: neutrophil. Black arrow: hepatocyte necrosis. Red arrow: hemorrhage. Scale bar: 50 μm. Representative histomorphometric images for the hematoxylin and eosin staining of liver tissue from each group. CF:* Corallodiscus flabellata *extract; WLP: Wu Ling Powder. L/D: Lipopolysaccharide and D-Galactosamine

**Figure 2:**
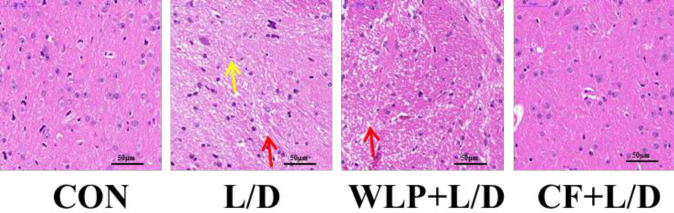
Effect of CF on L/D-induced pathological damage of brain tissue. WLP+L/D group (WLP, 63 mg/kg) and CF+L/D group (CF, 7000 mg/kg) were administered for 7 days, followed by intraperitoneal injection with L/D (10 μg/kg and 900 mg/kg). Red arrow: loose edema. Yellow arrow: nuclear pkynosis. Scale bar: 50 μm. Representative histomorphometric images for the hematoxylin and eosin staining of brain tissue from each group. CF: *Corallodiscus flabellata* extract; WLP: Wu Ling Powder. L/D: Lipopolysaccharide and D-Galactosamine

As illustrated in Figure 1, the histological analysis of the rat liver sections in the L/D group showed an obviously disturbed architecture, such as hepatocyte necrosis, hemorrhage, and neutrophil infiltration. However, the L/D-induced liver alterations were effectively relieved by the CF treatment. In addition, pathological changes in the brain were also detected. Figure 2 shows that the brain cells in the CON group were normal in morphology with no obvious edema. The brain cells in the L/D group were swollen and some of the nuclei were pkynosis or disappeared, with a tissue loose edema, and CF treatment can ease these symptoms.

***Effect of CF on ALT/AST and the levels of inflammatory factors***

**Figure 3:**
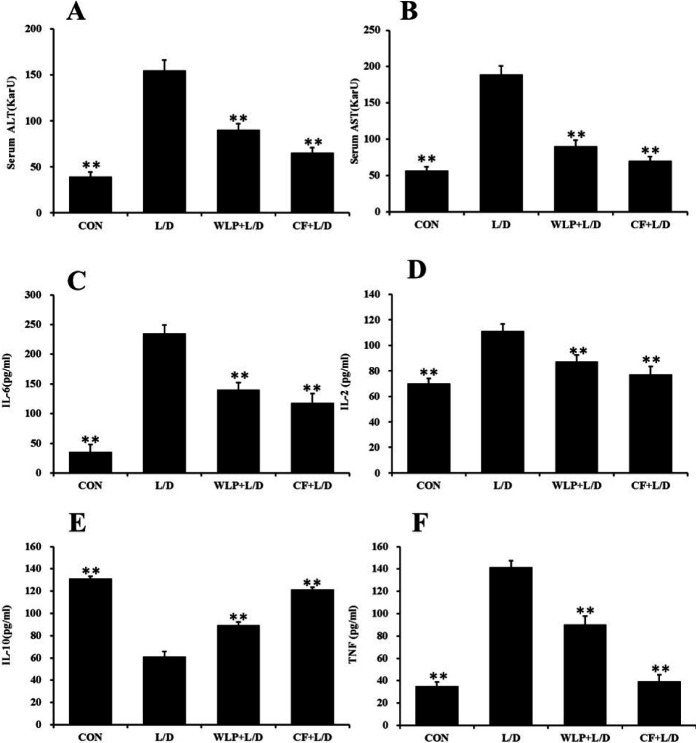
ALT/AST and the level of inflammatory factors. WLP+L/D group (WLP, 63 mg/kg) and CF+L/D group (CF, 7000 mg/kg) were administered for 7 days, followed by intraperitoneal injection with L/D (10 μg/kg and 900 mg/kg). (A) Effects on ALT. (B) Effects on AST. (C) Effects on IL-6. (D) Effects on IL-2. (E) Effects on IL-10. (F) Effects on TNF. All data are presented as means±SEM (n=8 in each group). *P< 0.05 and **P< 0.01 vs. L/D group. CF: *Corallodiscus flabellata* extract; WLP: Wu Ling Powder. L/D: Lipopolysaccharide and D-Galactosamine. ALT: Alanine aminotransferase. AST: Aspartate aminotransferase. TNF: Tumor necrosis factor. IL-2,6,10: Interleukin-2,6,10

ALT/AST is a representative indicator of the liver damage, while the inflammatory factors IL-6, IL-2, IL-10 and TNF play an important role in assessing the liver injury. This experiment investigated the effects of CF extract on serum ALT/AST and inflammatory factors in rats. The results are shown in Figure 3. Compared to the CON group, the levels of ALT/AST, IL-2, IL-6 and TNF in the L/D group were significantly increased (*P*< 0.01), while the levels of IL-10 were significantly lower (*P*<0.01). However, the ALT/AST content and IL-2, IL-6 and TNF levels were significantly low in the CF group (*P*<0.01), while the IL-10 levels were significantly high (*P*<0.01). The results show that CF has a certain liver protective function.

***Effect of CF on ROS content of primary hepatocytes and serum oxidative stress-related indexes***

**Figure 4:**
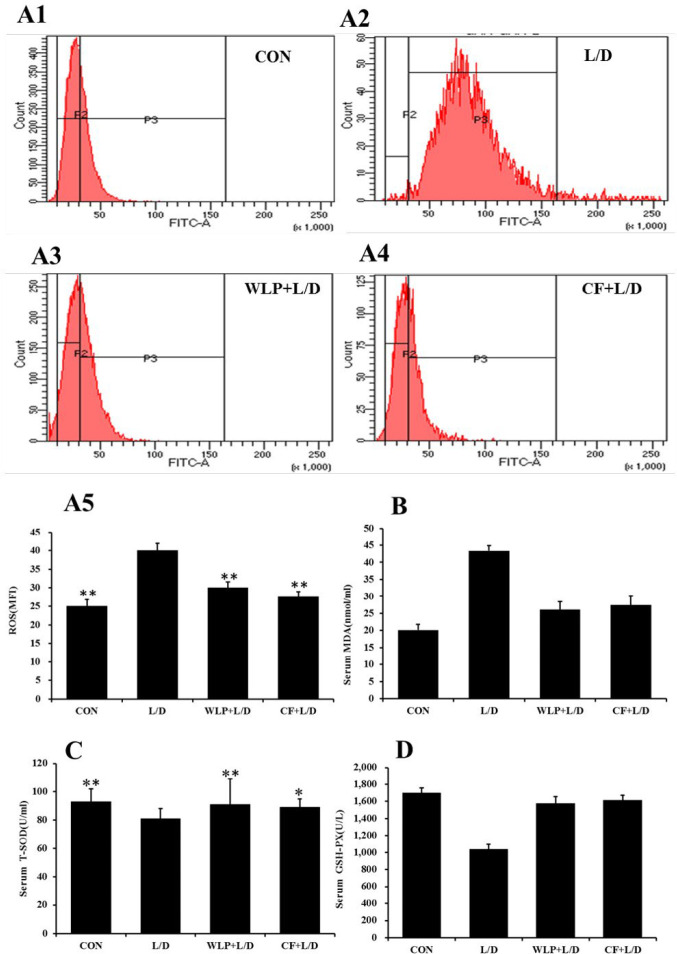
ROS content in rat primary hepatocytes. WLP+L/D group (WLP, 63 mg/kg) and CF+L/D group (CF, 7000 mg/kg) were administered for 7 days, followed by intraperitoneal injection with L/D (10 μg/kg and 900 mg/kg). (A) Effect on ROS. (B) Effect on MDA. (C) Effect on T-SOD. (D) Effect on GSH-PX. All data are presented as means±SEM (n=8 in each group). *P<0.05 and **P<0.01 vs. L/D group. CF: *Corallodiscus flabellata* extract; WLP: Wu Ling Powder. L/D: Lipopolysaccharide and D-Galactosamine. GSH-PX: Glutathione-peroxidase. MDA: Malondialdehyde. ROS: Reactive oxygen species. T-SOD: total superoxide dismutase

Oxidative stress is one of the key factors in the L/D-induced liver injury. Therefore, this experiment examined the effect of CF on the ROS content in primary hepatocytes and serum oxidative stress related indexes. The results are shown in Figure 4. L/D significantly raised the levels of ROS (*P*<0.01), and MDA (*P*<0.01) and reduced the levels of T-SOD (*P*<0.01), and GSH-PX (*P*<0.01), while CF reversed the changes induced by L/D.

***Effect of CF on rat liver apoptosis marker protein***

**Figure 5.**
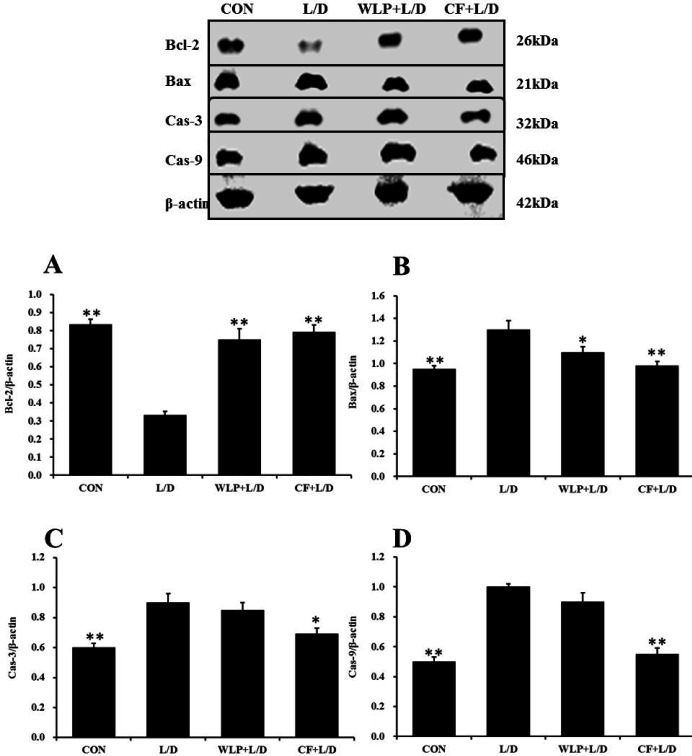
The expression of apoptotic marker protein in hepatocytes. WLP+L/D group (WLP, 63 mg/kg) and CF+L/D group (CF, 7000 mg/kg) were administered for 7 days, followed by intraperitoneal injection with L/D (10 μg/kg and 900 mg/kg). (A) Effect on Bcl-2. (B) Effect on Bax. (C) Effect on Caspase-3. (D) Effect on Caspase-9. All data are presented as means±SEM (n=3 in each group). *P<0.05 and **P<0.01 vs. L/D group. CF: *Corallodiscus flabellata* extract; WLP: Wu Ling Powder. L/D: Lipopolysaccharide and D-Galactosamine

A large number of literatures prove that L/D-induced liver damage is closely related to the hepatocyte apoptosis pathway. This experiment examined the effect of CF on markers of the hepatic apoptosis. As shown in Figure 5, the expression of Bcl-2 in the hepatocytes of the L/D group was significantly lower than that of the CON group (*P*<0.01), and the expression levels of Bax, Caspase-3 and Caspase-9 were significantly increased in L/D group (*P*< 0.01). CF increased the expression of Bcl-2 (*P*<0.01), and significantly reduced the expression levels of Bax (*P*<0.01), Caspase-3 (*P*<0.05) and Caspase-9 (*P*<0.01). Thus, CF inhibited the hepatocyte apoptosis induced by L/D.

***Effects of CF on the brain water content and expression of AQP1 and AQP4 in the brain***

**Figure 6.**
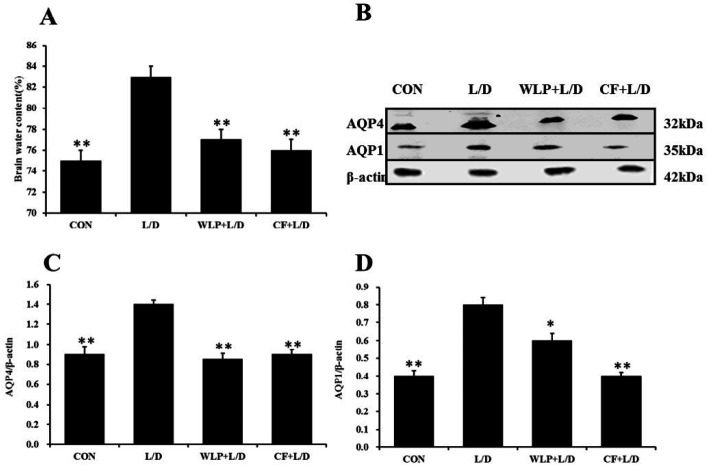
Brain water content and expression of AQP1 and AQP4 in the brain. WLP+L/D group (WLP, 63 mg/kg) and CF+L/D group (CF, 7000 mg/kg) were administered for 7 days, followed by intraperitoneal injection with L/D (10 μg/kg and 900 mg/kg). (A) Effects on brain water content. (B, C, D) Effects on AQP4 and AQP1. All data are presented as means±SEM (n=3 in each group). *P<0.05 and **P<0.01 vs. L/D group. CF: *Corallodiscus flabellata* extract; WLP: Wu Ling Powder. L/D: Lipopolysaccharide and D-Galactosamine

The pathological sections show that CF reduced the water content of brain tissue. Therefore, the water content of rat brain tissue was detected in this experiment, and the effects of CF on AQP1 and AQP4 in brain tissue were also examined. The results are shown in Figure 6. L/D enhanced the brain water content (*P*<0.01) and the expression of AQP1 (*P*<0.01) and AQP4 (*P *<0.01) in the brain (*P *<0.01). The brain water content (*P*<0.01) in the CF group and the expression levels of AQP4 (*P*<0.01) and AQP1 (*P*<0.01) were significantly lower than that of the L/D group.

***Effects of CF on the serum and urinary physical indicators***

The effects of CF on the urine volume, ion concentration and osmolality in serum and urine were detected. The results are shown in Table 1. Compared to the CON group, the urine volume of the L/D group increased slightly but was not obvious, while the Cl^-^ content in the urine and the urine osmolality increased significantly (*P*<0.01). Compared to the L/D group, the urine volume of the CF group was significantly increased (*P*<0.01), but the content of Cl^-^ in the urine and the urine osmolality were significantly decreased (*P*<0.01). As shown in Table 2, the Cl^- ^content in the serum of the L/D group was significantly increased compared to the CON group (*P*<0.01), while the Cl^- ^content of the serum significantly decreased in the CF group compared to the L/D group (*P*<0.05).

**Table 1:** Urine volume and electrolyte concentration and osmolality

Group	Dose (mg/kg)					
		8 hr (ml/100g)	Na^+ ^(mmol/l)	K^+ ^(mmol/l)	Cl^-^ (mmol/l)	urine osmotic (moms)
Con	--	1.34±0.37	62.3±13.8	8.54±0.59	42.4±2.1**	515.40±78.07**
L/D	--	2.34±0.20	60.0±3.9	8.34±0.22	59.6±2.9	707.17±32.04
WLP+L/D	63	4.8±0.30**	61.4±7.5	8.30±0.60	46.0±9.8**	556.40±69.36**
CF+L/D	7000	5.1±0.31**	60.0±14.5	8.40±0.41	46.3±1.8**	538.00±123.57**

Eight hr urine volume and electrolyte concentration and osmolality. WLP+L/D group (WLP, 63 mg/kg) and CF+L/D group (CF, 7000 mg/kg) were administered for 7 days, followed by intraperitoneal injection with L/D (10 μg/kg and 900 mg/kg). Then, the rats were placed in metabolic cages to collect urine for 8 hr. All data are presented as means±SEM (n=8 in each group). *P<0.05 and **P<0.01 vs. L/D group. CF: *Corallodiscus flabellata *extract; WLP: Wu Ling Powder. L/D: Lipopolysaccharide and D-Galactosamine

**Table 2:** Serum electrolyte concentration and osmolality of acute liver failure rats

Group	Dose (mg/kg)				
		Na^+ ^(mmol/l)	K^+ ^(mmol/l)	Cl^－ ^(mmol/l)	Serum osmotic (moms)
Con	--	119.8±11.5	1.84±0.07	58.3±2.0**	310.83±22.23
L/D	--	121.2±12.0	1.84±0.05	65.5±5.3	327.50±16.03
WLP+L/D	63	123.8±6.0	1.85±0.05	64.5±3.2	308.17±15.31
CF+L/D	7000	120.4±8.6	1.84±0.04	60.8±2.7*	309.33±24.45

Serum electrolyte concentration and osmolality. WLP+L/D group (WLP, 63 mg/kg) and CF+L/D group (CF, 7000 mg/kg) were administered for 7 days, followed by intraperitoneal injection with L/D (10 μg/kg and 900 mg/kg). All data are presented as means±SEM (n=8 in each group). *P< 0.05 and **P<0.01 vs. L/D group. CF: *Corallodiscus flabellata* extract; WLP: Wu Ling Powder. L/D: Lipopolysaccharide and D-Galactosamine

## Discussion

ALF is a severe hepatic insufficiency caused by a variety of causes, often accompanied by symptoms of hepatic encephalopathy. Hepatic encephalopathy is the most serious complication of liver failure and has a very high mortality rate. CE is a serious central nervous system complication caused by ALF (6). CE caused by ALF develops very rapidly and may cause cerebral palsy (7), posing a huge risk to the human body. Nevertheless, even minor degrees of edema often exhibit severe adverse effects as they compromise the cerebral blood flow or result in compression of brain structures on account of the fixed volume of the rigid skull. In this experiment, we successfully constructed a rat model of ALF using LPS combined with D-GalN. HE staining showed that L/D could cause obvious necrotic hemorrhage and inflammatory cell infiltration in the liver and an obvious brain swelling, while CF pretreatment significantly improved this change induce by L/D.

Under normal physiological conditions, ALT and AST are mainly present in hepatocytes, and when liver cells are damaged, ALT and AST will be transferred into the blood in large amounts. In our experiments, CF was found to significantly reduce the serum ALT and AST levels, which indicates that CF can significantly improve liver damage. There is a fair evidence that L/D can induce macrophages to secrete a large number of pro-inflammatory factor (IL-2, TNF etc.), triggering an inflammatory response and causing a series of damage to the body (8, 9). LPS stimulates the macrophage secretion of the pro-inflammatory factor TNF and also secretes anti-inflammatory factor IL-10 (10). IL-10 can form negative feedback on pro-inflammatory factors synthesized by the macrophages, thereby inhibiting its excessive release and exerting the anti-inflammatory effects. In addition, IL-10 can also promote the hepatocyte repair and regeneration (11). It has been reported that inflammatory factors can cause swelling of primary cultured astrocytes, and treatment of these inflammatory factors with ammonia beforehand aggravated the cell swelling (12). In addition, research has found that recombinant alkaline phosphatase was attenuated the severity of liver dysfunction and brain edema associated with reduction in cytokines, chemokines, liver cell death and brain water (13). In summary, the inflammation plays an important role in brain edema caused by ALF. This experiment shows that CF could significantly reduce the level of IL-6, IL-2, and TNF in the serum compared to the L/D group, and significantly increased the level of IL-10, which indicates that CF can significantly inhibit inflammation. These results correspond to the improvement of brain edema by a pretreatment with CF.

Being the most metabolically active organ in the human body, the liver is susceptible to damage from a variety of pathogens, drugs, and toxins. Oxidative stress induced by ROS is one of the common pathophysiological basis of various types of liver injuries (14, 15). Moreover, oxidative stress can increase the production of MDA formation to further result in liver tissue damage (16). ROS and MDA are typical markers of oxidative stress, while SOD and GSH-PX are important antioxidant enzymes that form the first line of defense by preventing free radical formation (17). This experiment demonstrated that CF could significantly reduce the content of ROS in primary hepatocytes and the content of MDA in serum compared to the L/D group, and significantly increased the levels of SOD and GSH-PX in serum, which indicates that CF could significantly inhibit oxidative stress. These results also correspond with the results of liver pathological sections. Thus, CF pretreatment can improve the pathological changes of the liver caused by L/D.

It is well known that two typical death modes of cells are necrosis and apoptosis. Necrosis is a passive death pathway for cells to cope with the environmental stress, and apoptosis is a physiologically active cell suicide behavior regulated by genes, which is a programmed death. Apoptosis is controlled by molecules of the B-cell lymphoma/leukemia-2 (Bcl-2) family that include pro-apoptotic protein Bcl-2 associated X protein (Bax) and anti-apoptotic protein Bcl-2. When cells are stimulated, Bax moves from cytoplasm to mitochondria and changes mitochondrial permeability, resulting in cytoplasmic mitochondrial cytochrome C (Cyt C) release, which in turn activates caspase and culminates in apoptosis (18). Our experiments showed that CF pretreatment significantly reversed the increase in Bax expression and the decrease in Bcl-2 expression caused by L/D. Caspase can maintain normal body function by regulating cell death and inflammation. The caspase family (caspase-3 and caspase-9) can induce apoptosis (19). Our experiments showed that CF pretreatment significantly reversed the increase of caspase-3 and caspase-9 protein expression induced by L/D. This suggests that CF pretreatment may play a role in liver protection by regulating apoptosis.

AQP4 is the main water transporter in the brain and is involved in edema formation (20). AQP4 acts as a direct passageway for fluid movement between brain tissue and blood vessels (21). AQP4 is a two-way water transport channel, which is not only a channel for water to enter brain tissue, but also a main channel for water to drain from the brain tissue, thereby playing an important role in the removal of excess water from the brain. AQP4 gene knockout significantly reduced the cytotoxic CE caused by water intoxication, cerebral ischemia and brain trauma (22, 23). It has also been reported that the expression of AQP4 in brain tissue was significantly up-regulated in bacterial meningitis (24). AQP4 gene knocking out can reduce the brain edema of bacterial meningitis, reduce the increase of intracranial pressure and thereby reduce the mortality rate (25). Transgenic mice overexpressing AQP4 can accelerate the progression of brain edema (26). AQP1 is the first discovered aquaporin, which is mainly found in the apical membrane of the choroid plexus, and also exists in the heart muscle and kidney. Studies have shown that AQP1 can mediate more water influx into the brain, leading to increased intracranial pressure and brain cell death, indicating that AQP1 plays an important role in the mechanism of water toxicity (27). Studies have also found that AQP1 plays an important role in the production of cerebrospinal fluid and the maintenance of intracranial pressure (28). In this experiment, L/D can up-regulate the expression of AQP4 and AQP1 in brain tissue, and CF pretreatment significantly down-regulated the expression of AQP4 and AQP1. It is suggested that CF pretreatment could improve the brain edema via the regulation of AQP4 and AQP1 expression.

Studies have shown that AQP1 and AQP4 have a certain correlation with the production of urine. In this experiment, we found that the urine volume of the CF pretreatment group was significantly higher than that of the L/D group. The amount of urine produced is mainly determined by the balance between the rate of generation of urine and the rate of reabsorption of water and salt in a certain period of time. When the rate of generation of raw urine increases or the reabsorption of water and salt is blocked, the amount of urine will increase. The urine concentration function is mainly reflected in the urine concentration factor. It is generally measured indirectly by the urine osmotic pressure. In fact, the urine osmotic pressure depends on the total concentration of urine solutes and the reabsorbed sodium, potassium, chlorine, and water. For this reason, we measured the osmotic pressure of serum and urine and the ion concentration therein. The results showed that there was no significant change in serum osmotic pressure in each group, but the osmotic pressure of urine in the L/D group was significantly high, and CF pretreatment significantly reversed this change. In the detection of ions in serum and urine, it was found that L/D can significantly increase the concentration of Cl^-^ in serum and urine, and CF pretreatment significantly reduced the concentration of Cl^-^. It is suggested that CF preconditioning can improve brain edema and may be related to the regulation of electrolyte imbalance.

In the early stage, our laboratory analyzed the chemical constituents of the total extract of CF by HPLC-MS. Six compounds in the total extract of CF including three phenylethanoid glycosides and three flavonoid glycosides were identified using mass spectrometry (29). It was confirmed that the phenylethanoid glycosides and flavonoid glycoside are the main components in CF*. *Our future studies aim to explore the material basis for CF to improve ALF.

## Conclusion

We envisaged the effect of CF on L/D-induced brain edema model of liver failure, and further examined its mechanism of action to confirm its effectiveness. It was observed that CF could improve the ALF and brain edema induced by L/D by inhibiting the hepatocyte apoptosis, inflammation, oxidative stress and brain AQP1, and AQP2 levels.
